# A Medical Causerie

**Published:** 1915-07-10

**Authors:** 


### A MEDICAL CAUSERIE.

RUMOURS have been current that the Government intend shortly to mobilise the whole body of the profession and to appoint to each individual member his own particular share of patriotic duty. It is quite impossible to put any faith in such a story. What may happen in the course of the struggle in which the nation is at present engaged no man may say; but it is impossible to believe that there is any substance in the report that a particular body of citizens is to be put under compulsion while others remain free in their choice. Further, according to a recent Government statement in the House of Commons, the supply of medical men, so far as the immediate wants of the Army are concerned, is not inadequate. The point of anxiety arises when the future is contemplated. As more men take the field more doctors will be wanted; the question is, Will they be forthcoming? There are professional organisations at work for the purpose of securing an affirmative reply to this question, and considerable progress has been made. Some months ago the Director-General made a moving appeal for support, but since then he has kept silence. Whether he has obtained all that he requires is one of the numerous secrets which a tender and considerate War Office thinks it well to withhold from a shrinking and sensitive British public.

Much satisfaction is expressed at the award of the Lecomte Prize to Sir Almroth Wright by the Paris Académie des Sciences. The prize is adjudged biennially, and is of the value of £2,000. The precise, practical, and scientific quality of Sir Almroth's work doubtless makes a special appeal to the logical French mind. It has now been tested on a large scale, and though the end is not yet, a considerable body of achievement is secure beyond challenge. Possibly the Paris Académie does not read certain enthusiastic publicists' communications, or perchance the award might have taken a different direction.

Criticism of the Admiralty arrangements by which a group of consulting physicians and surgeons were retained for official service, each at a salary of £5,000, has apparently "touched the spot," as our readers will appreciate from the important article which appears on page 309 this week. The payment of such substantial salaries was not easily defended, seeing that corresponding duties discharged for the Army were secured for sums little more than one-quarter that paid by the Admiralty. At the same time, it must be recognised that the civil physicians and surgeons engaged on active service in a consultant capacity with the forces in France and the Near East, and holding the rank of colonel, have accepted the positions they hold not for business, but for patriotic reasons. Most of them, if not all, must be making very considerable pecuniary sacrifices. The salaries paid to them are far below their usual professional incomes; their absence from London and other cities where they practise means the risk of loss of connection and of advance; and with all this they have to continue the expenses which are a necessary part of their professional status. If it be said that the soldiers who hold a corresponding rank are in the same position, an adequate reply is easily found. First, the soldiers are under no necessity to keep up expensive houses at home; speaking generally, they are younger men, and they hope for and expect promotion to higher ranks and more substantial pay; and their present pay, such as it is, is part of their professional bargain, and does not involve the deliberate sacrifice of a larger income from other sources. Still further, it has to be remembered that the doctors are selected because it is believed that they are at the very head of their profession, and hence they might claim to stand on the same platform as the heads of the Army. There are many colonels who never rise to be generals, and equally there are many consulting physicians and surgeons who never attain the front rank. In both cases when the exceptions were selected, the selection according to current practice commanded a corresponding measure both of position and pay. We have shown on page 309 how both the Admiralty and the Army can continue to command the best medical and surgical advice and assistance whatever the pay and whatever the rank. This is secured alike by the needs of the country and by the circumstances of the profession. In some quarters adverse remarks are being made on the much-increased salaries now asked by the junior members of the profession, but the large sacrifices made in other directions are forgotten. The losses suffered by many of the medical officers in the Territorial Forces have been in many instances disastrous, and generally the voluntary and unpaid work of the profession has been greatly increased by the war.

It is a trite remark that science does not pay, and there would seem to be justification for it in the recent award of Civil List Pensions. The list includes the names of two Fellows of the Royal Society, one being a highly accomplished physician and the other the author of valuable researches in physics and physiology. To read of "straitened circumstances" and "inadequate means of support" in this connection is a painful experience, and the list shows that these conditions may even fall upon the family of a man whose celebrity entitled him to write O.M. after his name.

The King Edward Sanatorium at Midhurst, which, early in the present year, and at the request of the War Office, set aside fifty beds for soldiers suffering from pulmonary tuberculosis, is now free to resume its special purpose—namely, the reception of male and female patients who belong to the educated classes and whose means are limited. The non-commissioned officers and men are for the most part beneficiaries under the National Insurance Act, and the Commissioners have made arrangements for their treatment in sanatoria in the localities where they propose to reside.

